# Pro-osteoporotic miR-320a impairs osteoblast function and induces oxidative stress

**DOI:** 10.1371/journal.pone.0208131

**Published:** 2018-11-28

**Authors:** Laura De-Ugarte, Susana Balcells, Xavier Nogues, Daniel Grinberg, Adolfo Diez-Perez, Natalia Garcia-Giralt

**Affiliations:** 1 Department of Anatomy and Cell Biology, Indiana University School of Medicine, Indianapolis, Indiana, United States of America; 2 Indiana Center for Musculoskeletal Health, Indianapolis, Indiana, United States of America; 3 Department of Genetics, Microbiology and Statistics, Facultat de Biologia, Universitat de Barcelona, Centro de Investigación Biomédica en Red de Enfermedades Raras (CIBERER), ISCIII, IBUB, IRSJD, Barcelona, Catalonia, Spain; 4 Musculoskeletal Research Group, IMIM (Hospital del Mar Medical Research Institute), Centro de Investigación Biomédica en Red en Fragilidad y Envejecimiento Saludable (CIBERFES), ISCIII, Barcelona, Catalonia, Spain; Universite de Nantes, FRANCE

## Abstract

MicroRNAs (miRNAs) are important regulators of many cellular processes, including the differentiation and activity of osteoblasts, and therefore, of bone turnover. MiR-320a is overexpressed in osteoporotic bone tissue but its role in osteoblast function is unknown. In the present study, functional assays were performed with the aim to elucidate the mechanism of miR-320a action in osteoblastic cells. MiR-320a was either overexpressed or inhibited in human primary osteoblasts (hOB) and gene expression changes were evaluated through microarray analysis. In addition, the effect of miR-320a on cell proliferation, viability, and oxidative stress in hOB was evaluated. Finally, matrix mineralization and alkaline phosphatase activity were assessed in order to evaluate osteoblast functionality. Microarray results showed miR-320a regulation of a number of key osteoblast genes and of genes involved in oxidative stress. Regulation of osteoblast differentiation and ossification appeared as the best significant biological processes (PANTHER P value = 3.74E-05; and P value = 3.06E-04, respectively). The other enriched pathway was that of the cellular response to cadmium and zinc ions, mostly by the overexpression of metallothioneins. In hOBs, overexpression of miR-320a increased cell proliferation and oxidative stress levels whereas mineralization capacity was reduced. In conclusion, overexpression of miR-320a increased stress oxidation levels and was associated with reduced osteoblast differentiation and functionality, which could trigger an osteoporotic phenotype.

## Introduction

MicroRNAs (miRNAs) are considered important regulators of cellular processes related to bone metabolism, among others. A number of miRNAs have been reported to regulate the differentiation and activity of osteoblasts and osteoclasts by targeting genes with a key role in bone turnover [1], although the full mechanism of action remains unknown.

Identifying the specific functions of miRNAs in bone may give insights into the pathophysiology of skeletal disorders. For example, a microarray analysis has shown overexpression of miR-320a in whole bone tissue from women with osteoporotic fracture [2, 3]. Moreover, miR-320a is elevated in osteoarthritic cartilage tissue and chondrocytes [4], highlighting its potential functionality in the musculoskeletal system. This miRNA has been reported to have multiple mRNA targets and to regulate different pathways in various cell types. To date, most studies on miR-320a involve cancer malignancies [5–8], followed by other pathologies such as cardiovascular diseases [9, 10] or Type II diabetes [11, 12]. In bone tissue, miR-320a is expressed in human primary osteoblasts (hOBs) and in differentiated osteoclasts (hOCs) and is predicted to regulate genes involved in bone metabolism [2, 13]. Recently, Huang et al. [14] demonstrated that miR-320a inhibits the osteogenic differentiation of mesenchymal stem cells by targeting *HOXA10*. This is consistent with a previous study showing downregulation of miR-320a during osteogenic differentiation of fibroblasts *in vitro* [15]. However, the role of miR-320a in the context of bone and in differentiated osteoblasts has not been explored.

In the present study, we performed functional assays with the aim to elucidate the mechanism of miR-320a action in osteoblastic cells. For this purpose, miR-320a was either overexpressed or inhibited in hOB and gene expression changes were assessed through microarray analysis. In addition, osteoblast functionality was evaluated by assessing matrix mineralization and alkaline phosphatase (ALP) activity. Finally, the effect of miR-320a on cell proliferation, viability, and oxidative stress was evaluated.

## Materials and methods

### Cell culture

Human primary osteoblasts (hOBs) were obtained from fresh trabecular bone from postmenopausal women subject to a knee transplant surgery due to osteoarthritis. Exclusion criteria were any history of metabolic or endocrine disease, chronic renal failure, chronic liver disease, malignancy, Paget’s disease of bone, malabsorption syndrome, and any bone metabolism-affecting treatment. The study was carried out in accordance with the Declaration of Helsinki, and the approved protocol for obtaining primary osteoblasts from knee samples otherwise discarded at the time of orthopedic surgery was explained to potential participants. Written informed consent was obtained from all individual participants included in the study. The Clinical Research Ethics Committee of Parc de Salut MAR approved the present research (Registry number 2010/3882/I and 2013/5266/I).

Bone samples were carefully obtained from a location distant from the interface between bone and cartilage and, therefore, as far away as possible from the osteoarthritic lesion. Bone tissue was cut up into small pieces, washed in phosphate buffered solution (PBS, Gibco by Life Technologies; Paisley, UK) to remove non-adherent cells, and cultured on a 140mm culture plate with Dulbecco’s Modified Eagle Medium (DMEM; Gibco; Invitrogen, Paisley, UK) supplemented with 10% fetal bovine serum (FBS; Sigma-Aldrich; St. Louis, USA), 100 U/ml penicillin/streptomycin (Sigma Aldrich; St. Louis, USA), 0.4% fungizone (Gibco by Life Technologies; Paisley, UK), and 100 μg/ml ascorbic acid (Sigma-Aldrich; Steinheim, Germany). Bone samples were discarded if patients had a medical history of using oral corticosteroids, anti-resorptive or anabolic agents, anti-epileptic drugs, lithium, heparin or warfarin, or a diagnosis of chronic renal failure, chronic liver disease, malignancy, or metabolic or endocrine diseases affecting bone. HOBs were cultured in tissue flasks in DMEM supplemented with 10% FBS, 100 U/ml penicillin/streptomycin, and 100 μg/ml ascorbic acid (osteoblastic medium) until the required number of cells was reached.

Five hOB samples were used for the gene expression microarray, three independent samples were used for microarray validation by real time PCR, and another six samples were used for the osteoblast functional assays. All experiments were performed at maximum passage 2.

### Cell transfection

Cells were seeded on different well plates depending on the type of experiment to be carried out after cell transfection. For microarray assay, a 6-well plate with 180,000 cells/well was used. Two wells were cultured in parallel for qPCR validation of microarray results. To assess cell viability (MTS), cell proliferation (BrdU), alkaline phosphatase (ALP) activity, and CellRox Green Reagent assay, a 96-well plate with 12,000 cells/well was used. Finally, a 24-well plate with 45,000 cells/well was used to perform Alizarin Red assays.

Cells at 70–80% of confluence were transfected with mirVana mimic (M) or inhibitor (I) of hsa-miR-320a, using Lipofectamine RNAiMAX (Invitrogen; Carlsbad, USA) according to manufacturer instructions, with the exception of 20 minutes of incubation time for miRNA -Lipofectamine interaction instead of 5 minutes. MirVana miRNA Mimic Negative Control #1 (CM) and mirVana miRNA Inhibitor Negative Control #1 (CI), respectively, were used as controls. All products were purchased from Ambion Life Technologies (Madrid, Spain). Mimic and control mimic were used at 100 nM and inhibitor and control inhibitor at 400 nM. In order to monitor transfection efficiency, miRIDIAN microRNA Mimic Transfection Control with Dy547 (Dharmacon, 100 nM) was transfected at the same conditions.

Cells transfected with the Mimic Transfection Control with Dy547 were stained with 4',6-diamidino-2-phenylindole (DAPI) dihydrochloride **(**0’2mg/ml) (Sigma-Aldrich) 24 hours after transfection to distinguish the nucleus of the cell. Then, cells were observed through the LEICA DMIL LED fluorescence microscope using the Leica Application Suite (Leica Microsystems).

### MiR-320a quantification by quantitative real time PCR (qPCR)

To evaluate the post-transfection miR-320a expression levels in hOBs, total RNA was extracted 48 hours after transfection using the miRNeasy mini kit (Qiagen) according to manufacturer instructions. Then, 1 μg of total RNA was reverse-transcribed in 20 μl reactions using the miScript II RT kit (Qiagen). cDNA was diluted 1/8 and 2 μl were assayed in 10 μl qPCR reactions in 384-well plates using MiScript SYBR Green PCR kit according to the protocol. The mature miR-320a sequence, according to the mirBase web site, was used as a forward primer (5’-AAAAGCTGGGTTGAGAGGGCGA-3’) and the Universal primer as a reverse. U6 snRNA was used as the reference gene for normalization. All qPCR reactions for each sample were performed in triplicate. Amplification was performed in a QuantStudio 12K Flex Real-Time PCR (Applied Biosystems), and the ExpressionSuite software v.1.0.3 (Life Technologies) was used both for determination of relative quantification (RQ) (by 2-ΔΔCt method) and for melting curve analysis.

### Gene expression microarray analysis

Changes in gene expression levels at 48 hours after transfection of hOBs (n = 5) with miR-320a mimic or inhibitor, as well as their respective controls, were measured by microarrays. Total RNA from hOBs samples was obtained using the RNeasy mini kit (Qiagen) according to manufacturer instructions. RNA was assayed by IMIM Microarray Analysis services (Institut Hospital del Mar d'Investigacions Mèdiques). RNA integrity was assessed using Agilent 2100 Bioanalyzer (Agilent Technologies). All samples met the quality standards (RNA integrity number (RIN) >7; Ratio 260/280 > 1,6) and were used in microarray experiments. Amplification, labeling, and hybridizations were performed according to protocol with GeneChip WT PLUS Reagent kit (Affymetrix) and then hybridized to GeneChip Human Gene 2.0 ST Array in a GeneChip Hybridization Oven 640. Washing and scanning were performed using the Expression Wash and Stain and the Affymetrix GeneChip System (Fluidics Station 450 and Scanner 3000 7G). After quality control of raw data, data were background-corrected, quantile-normalized, and summarized to a log2 gene level using the robust multi-chip average (RMA), obtaining a total of 48,144 transcript clusters, excluding controls, which roughly correspond to genes or other mRNAs such as miRNAs or lincRNAs.

The Log2 Fold Change (Log2FC) was calculated for each sample and gene as ΔM = CM–M and ΔI = I–CI. The order of the factors in the subtractions was inverted, given that the expected effect of Mimic and Inhibitor are reverse. Data analyses were performed in R (v 3.1.1) with the aroma.affymetrix package. First analysis selected those genes differentially expressed between mimic and control mimic samples (ΔM) or between inhibitor and control inhibitor (ΔI) with a Log2FC >1.5 and P value <0.05, calculated by moderated t-test [16]. The second analysis selected those genes differentially expressed in both comparisons (ΔM and ΔI) with a Log2FC >1.2 and P value <0.05 and, moreover, overlapped in both ΔM and ΔI profiles.

Genes differentially expressed (upregulated and downregulated) underwent a further functional analysis using the Database for Annotation, Visualization and Integrated Discovery (*DAVID*) v6.8 [17], PANTHER Classification System [18] and the GeneMANIA prediction server (http://www.genemania.org) [19].

### Gene expression quantification with real time PCR (qPCR)

At 48 hours post-transfection, total RNA extraction of hOBs (n = 3) was performed using the RNeasy mini kit (Qiagen) according to manufacturer instructions. An additional DNase I treatment was performed to avoid genomic contamination.

RNA quantity and purity was determined on the ND-2000 Spectrophotometer (NanoDrop Technologies). Samples for the microarray were stored at -80°C until use, while samples for the qPCR were stored at -20°C.

For qPCR assays, 200 ng of total RNA were retrotranscribed to cDNA following the instructions of the High-Capacity cDNA Reverse Transcription Kit (Life Technologies). Product was diluted 1/10 and 2 μl were assayed in 10 μl PCR reactions in 384-well plates using commercially available TaqMan Gene Expression assays (Life Technologies). Gene expression levels were calculated against beta-actin expression and then normalized to an internal sample (relative quantification) using arbitrary units.

### Cell viability assay (MTS)

Viable cells were determined 48 hours after miR-320a transfection in hOBs (n = 3) using the CellTiter 96 AQueous One Solution Cell Proliferation Assay (Promega; WI, USA) according to manufacturer instructions. To measure the amount of soluble formazan produced by cellular reduction of MTS, the absorbance at 490 nm was measured using a multi-well plate reader Infinite M200 (Tecan; Grödig, Austria).

### Cell proliferation quantification

Proliferation was quantified after 48 h from miR-320a transfection in hOBs (n = 3) based on the measurement of BrdU incorporation during DNA synthesis with Cell Proliferation Elisa, BrdU (Colorimetric) (Roche).

### Alizarin red quantification

To quantify mineralization in hOBs (n = 6), Alizarin red assay was performed after 28 days of culture. Cells were transfected with both mimic and inhibitor of miR-320a and the corresponding controls at day 1 and day 14 after seeding.

Cells were cultured with osteoblastic medium supplemented with 5mM β-glycerophosphate (Sigma-Aldrich, St Louis, MO, USA); the medium was changed twice per week. At 28 days, media was removed from the cell monolayer and gently washed three times with PBS. Then, cells were fixed in 10% buffered formalin for 10 minutes at room temperature. The fixative was removed and cultures washed with PBS. The cell layer was stained with 2% Alizarin-S (Sigma- Aldrich, St Louis, MO, USA) at ~pH 4.2 for 20 minutes. Cell preparations were washed with PBS to eliminate nonspecific staining.

To measure calcium deposition, the dye was leached from the monolayer by the addition of 10% cetylpyridinium chloride until all of the dye had been drawn from the monolayer. At that point, 100 μl of the solution was transferred to a clean 96-well plate for quantifying spectrophotometry at 550 nm (using 10% cetylpyridinium chloride as a blank reference).

### ALP activity assay

ALP activity was measured in hOBs (n = 3) at 48 hours after miR-320 transfection using the Alkaline Phosphatase Assay Kit (Colorimetric) (Abcam; Cambridge, UK) according to manufacturer instructions.

### CellRox Green Reagent assay

Cellular oxidative stress was tested with CellRox Green Reagent (10μM) in 96-well plates at 72 hours after transfection, following manufacturer instructions. Immediately, CellRox Green Reagent fluorescence was evaluated through the LEICA DMIL LED fluorescence microscope using the Leica Application Suite (Leica Microsystems). A fluorescence comparison between mimic and control mimic, inhibitor and control inhibitor was performed using ImageJ (Image Processing and Analysis in Java) from NIH.

### Statistical analysis

Mann-Whitney U test in the SPSS v.12.0 for Windows was performed to establish comparisons between cells transfected with miRNAs and their respective controls in the Alizarin Red quantification, alkaline phosphatase activity, cell proliferation assay and detection of miRNA and mRNA levels by qPCR. All analyses were two-tailed, and p-values <0.05 were considered significant.

## Results

### Gene expression analysis after miRNA-320a mimic/inhibitor transfection in osteoblasts

In order to evaluate the effect of miR-320a on hOB gene expression, a gene microarray analysis was performed after overexpressing or inhibiting the miR-320a (available in GEO; GSE121892). As a first step, the efficiency of miRNA transfection was monitored using miRIDIAN microRNA Mimic Transfection Control with Dy547 (S1A Fig) and by determining miR-320a levels after mimic or inhibitor transfection (S1B Fig). Results demonstrated a high transfection efficiency by increasing the miR-320a levels more than 400-fold after mimic transfection (p = 0.002) and a marked downregulation to residual levels by the inhibitor transfection (p = 0.002)

The microarray analysis, using a Log2FC >1.5 and P value <0.05, identified 57 genes differentially expressed in control mimic vs. mimic samples (ΔM). These genes are shown in a hierarchical clustering heat map (S2 Fig); those with UCSC annotations are shown in Table 1 (n = 38).

**Table 1:** Differentially expressed genes for ΔM with UCSC annotation.

UCSC symbol	LogFC	P Value
*5S_rRNA*	0,74	0,0003
*U6*	0,73	0,0001
*AK056897*	0,72	0,0047
*GBP1*	0,69	0,0377
*GDF5*	0,67	0,0028
*TGOLN2*	0,64	0,0000
*AK097957//CDCA3*	0,62	0,0148
*abParts*	0,61	0,0005
*MIR548Y*	0,61	0,0056
*SYNGR2*	0,60	0,0001
*Y_RNA*	0,59	0,0141
*MIR934//VGLL1*	-0,58	0,0029
*IFT80*	-0,58	0,0308
*IER3*	-0,59	0,0117
*BC065763*	-0,61	0,0022
*MIR31//IFNE//MIR31HG*	-0,61	0,0005
*DOCK4*	-0,62	0,0035
*MT1X*	-0,64	0,0028
*PGM2L1*	-0,64	0,0448
*JARID2*	-0,64	0,0495
*DKK2*	-0,66	0,0001
*ITGA2*	-0,66	0,0066
*MT1G*	-0,66	0,0010
*IGFBP1*	-0,69	0,0236
*BC045559//SLC6A15*	-0,70	0,0039
*CDKN2B*	-0,71	0,0091
*AREG*	-0,71	0,0360
*BMP2*	-0,72	0,0121
*RORB*	-0,72	0,0006
*MT1F*	-0,74	0,0019
*LIF*	-0,77	0,0408
*SLC16A6*	-0,78	0,0001
*SMOC1*	-0,80	0,0025
*MET*	-0,82	0,0006
*NPTX1*	-0,82	0,0011
*MIR4279*	-0,84	0,0222
*RANBP3L*	-0,85	0,0037
*PTGS2*	-0,97	0,0395

To validate these results, 3 genes (*BMP2*, *PTGS2*, and *IGFBP1*), chosen by known importance in osteoblastic function, were analyzed using qPCR. All three were confirmed: they showed significant differences after mimic transfection and in the same direction as that observed in the microarray (S3 Fig).

The same analysis found 41 genes differentially expressed in inhibitor vs. control inhibitor (ΔI) samples, with a Log2FC >1.5 and P value <0.05, shown in a hierarchical clustering heat map (S4 Fig); 23 of these genes had UCSC annotations (Table 2). In both heat maps, samples were grouped according to the transfected RNA (mimic or control, inhibitor or control).

**Table 2:** Differentially expressed genes for ΔI with UCSC annotation.

UCSC symbol	LogFC	P Value
*PRKDC*	0,84	0,0040
*SNORD41//TNPO2*	0,79	0,0021
*TESK2*	0,75	0,0000
*CR936796*	0,73	0,0110
*SGMS1*	0,72	0,0042
*ZNF876P*	0,70	0,0021
*MAPK6*	0,67	0,0417
*MIR1234//CPSF1*	0,67	0,0026
*TPTE*	0,67	0,0057
*RHOU*	0,64	0,0005
*SYNPR*	0,61	0,0011
*KLHL2*	0,60	0,0105
*MIR3065*	0,60	0,0095
*EPS8*	0,59	0,0012
*MIR323A*	0,58	0,0387
*FLJ41484//GAS6-AS1*	0,58	0,0002
*ULK4P3*	0,58	0,0056
*VTRNA1-2*	-0,58	0,0437
*FPR3*	-0,61	0,0122
*SNORD115-45//SNURF-SNRPN*	-0,62	0,0018
*SOD2*	-0,63	0,0003
*KRTAP9-3*	-0,69	0,0047
*TULP3*	-0,87	0,0004

Since no gene was found in both sample clusters (ΔM and ΔI), we performed an additional analysis, decreasing the Log2FC to >1.2. Using this new threshold, a total of 80 genes overlapped the ΔM and ΔI profiles. Of these, the 32 genes with UCSC annotations are listed in Table 3.

**Table 3:** Overlapped genes between ΔM and ΔI profiles with UCSC annotation. The LogFC is >1.2 and p value <0.05.

UCSC symbol	LogFC ΔM	P Value ΔM	LogFC ΔI	P Value ΔI
*5S_rRNA*	0,74	0,0003	0,37	0,0415
*abParts*	0,61	0,0005	0,43	0,0097
*AK308704//TMEM191C*	0,37	0,0133	0,32	0,0323
*AKR1C3*	-0,34	0,0084	-0,37	0,0044
*BC068543//GBF1*	0,36	0,0423	0,37	0,0386
*BHLHE23*	0,36	0,0179	-0,30	0,0431
*BTN3A3*	0,48	0,0066	0,40	0,0195
*C10orf107*	0,42	0,0016	0,28	0,0258
*CCDC30*	0,39	0,0203	0,39	0,0185
*CKMT1A//RNU6-28P*	-0,40	0,0067	0,31	0,0325
*CSH2*	0,26	0,0450	-0,34	0,0121
*CYP4F30P*	-0,33	0,0292	-0,33	0,0315
*DKFZp667J0810*	0,30	0,0196	0,30	0,0194
*FLJ41484//GAS6-AS1*	0,29	0,0359	0,58	0,0002
*INSM2*	0,26	0,0248	-0,31	0,0093
*LRP8*	-0,32	0,0350	-0,31	0,0389
*LRRC37A3*	0,56	0,0032	0,35	0,0492
*MIR501//CLCN5*	-0,51	0,0017	-0,42	0,0073
*NAA15*	-0,31	0,0070	0,27	0,0153
*OR7E91P*	0,45	0,0187	0,44	0,0232
*PABPC1*	0,41	0,0264	0,42	0,0229
*PTPN1*	0,46	0,0369	-0,52	0,0201
*SLC2A13*	-0,37	0,0258	-0,40	0,0164
*SLC35G2*	-0,30	0,0152	-0,31	0,0135
*SPAG11A//SPAG11B*	0,26	0,0356	0,27	0,0335
*SPDYE5*	-0,45	0,0366	0,44	0,0406
*TESK2*	0,30	0,0265	0,75	0,0000
*TFPT*	0,35	0,0129	0,27	0,0437
*TRAV12-1//TCRA//TRA*	0,46	0,0024	0,36	0,0141
*U3*	-0,53	0,0155	-0,54	0,0142
*U6*	-0,47	0,0196	-0,47	0,0197
*ULK4P3*	0,54	0,0092	0,58	0,0056

**Fig 1:**
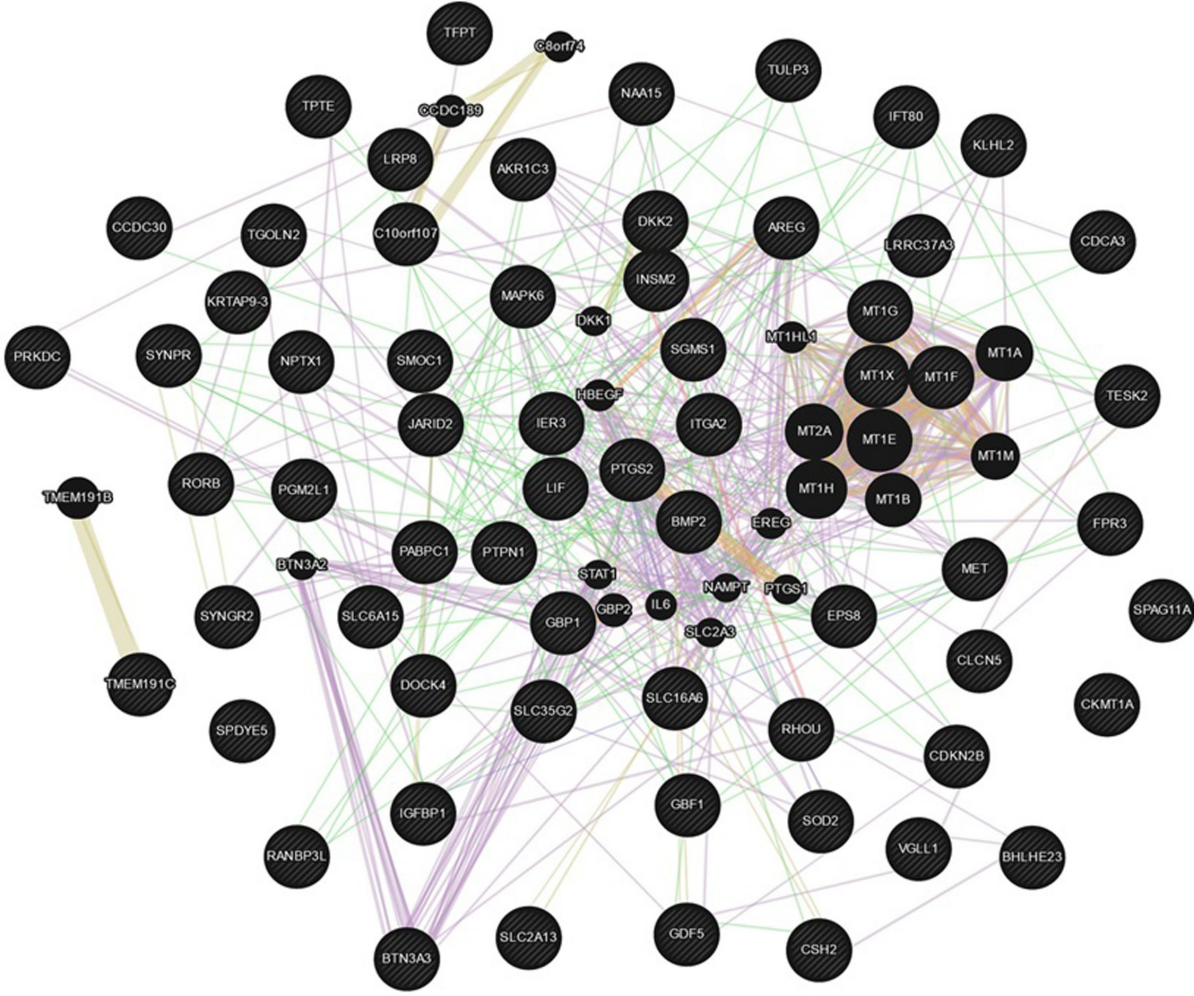
Functional interaction network from miR-320a-regulated genes plus the genes most related to the original list using GeneMANIA prediction server.

DAVID tools and PANTHER overrepresentation test analyzing genes from Tables 1, 2 and 3 detected the best enrichment scores in the cellular response to cadmium (P value = 2.07E-05) and zinc ions (P value = 1.67E-3), which are related to negative regulation of growth (DAVID enrichment score = 2.8) as well as an enrichment in growth factor and cytokine activity (P values = 0.014 and 0.017, respectively) with a DAVID enrichment score of 1.5. Regulation of osteoblast differentiation and ossification appeared as best significant biological processes (PANTHER P value = 3.74E-05; and P value = 3.06E-04, respectively). Mesenchymal cell differentiation involved in bone development, age-dependent response to oxidative stress, and positive regulation of Wnt signaling pathway by BMP raised among the top listed fold enriched pathways in PANTHER analysis. Genemania analysis corroborated these functions and moreover showed a network containing key genes for bone metabolism: *LIF*, *DKK1*, *DKK2*, *BMP2*, *GDF5*, *IGFBP1*, *IL-6*, etc. (Fig 1).

Additionally, key transcription factors involved in the osteoblast differentiation were assessed by qPCR after transfection with miR-320a mimic or inhibitor and their respective controls. Among them, *HOXA10* [14], *RUNX2* [20], and *Beta-Catenin* [21] are direct targets of miR-320a. *HOXA10* and *RUNX2* genes were confirmed in our study as targets of miR-320a even though differences in *RUNX2* gene expression did not reach significant levels (S5 Fig).

### Cell viability and proliferation

**Fig 2:**
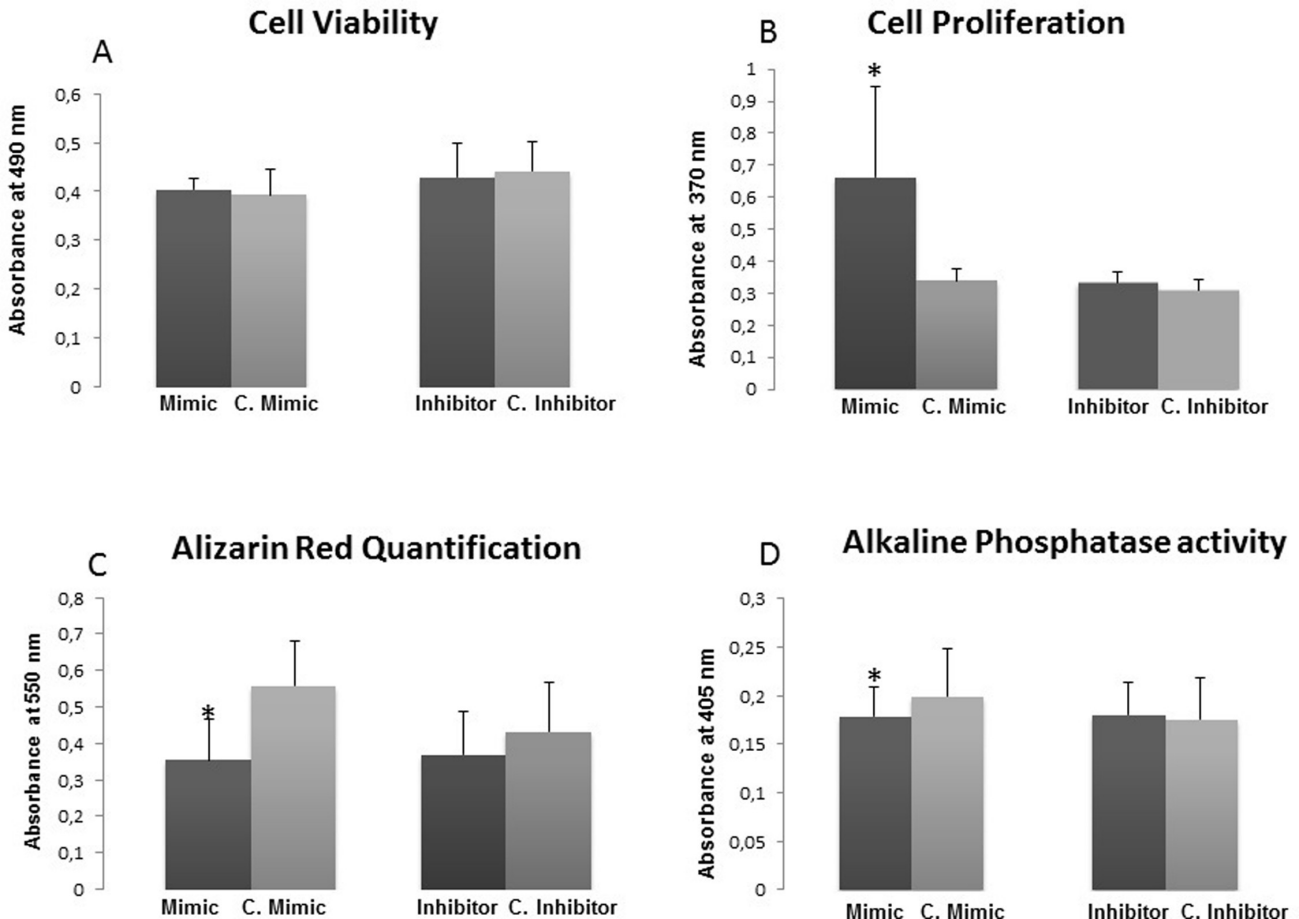
Effect of miR-320a on human osteoblast cell function. (a) cell viability, (b) proliferation, (c) Alizarin Red quantification, and (d) alkaline phosphatase activity were determined in primary hOBs (n = 6) transfected with mimic and inhibitor of miR-320a and its respective controls. Data represent the mean ± SD. *p<0.05.

Primary osteoblast viability and proliferation were assessed after 48 h from transfection with miR-320a mimic or inhibitor and the respective controls. Neither the transfection of miR-320a mimic nor that of the inhibitor caused significant changes in cell viability (Fig 2A). On the other hand, overexpression of miR-320a increased hOB proliferation (P = 0.045) (Fig 2B).

### Osteoblastic function

Osteoblast functionality after transfection was evaluated through Alizarin red staining and alkaline phosphatase activity quantification. Overexpression of miR-320a significantly reduced matrix mineralization of hOBs (P = 0.02) (Fig 2C) and ALP activity (P = 0.038) (Fig 2D).

Moreover, two crucial proteins of bone extracellular matrix were assessed by qPCR after miR-320a transfection. Gene expression of both genes, *COL1A1* and *BGLAP*, seems to be slightly affected by miR-320a (S5 Fig).

### Cellular oxidative stress assessment

**Fig 3:**
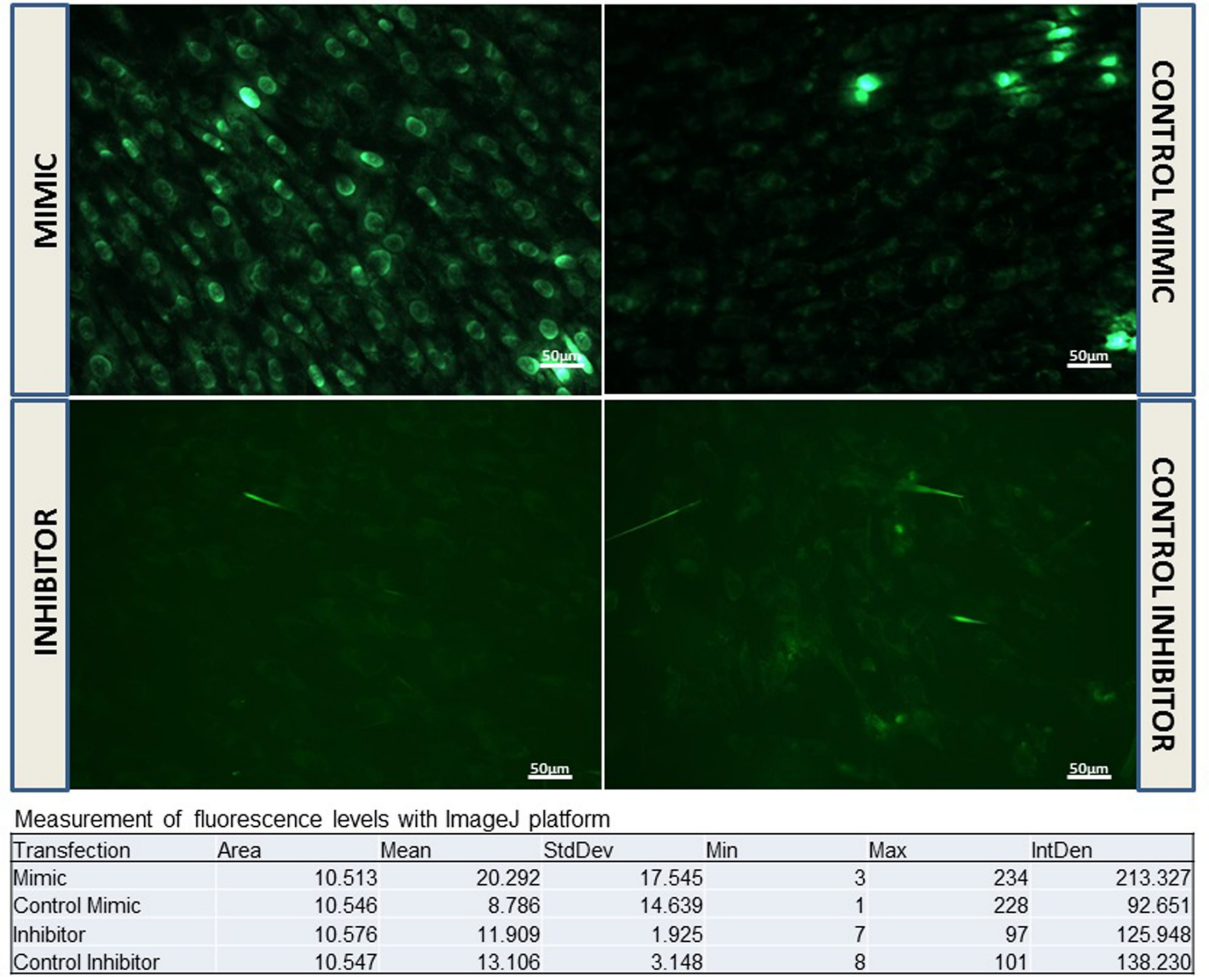
Cellular oxidative stress was tested using CellRox Green Reagent at 72 hours after miR-320a mimic/inhibitor transfection. Fluorescence was evaluated with the LEICA DMIL LED fluorescence microscope and with the Leica Application Suite (Leica Microsystems).

Reactive oxygen species (ROS) were detected in hOB cells using CellRox Green Reagent at 72 hours after miR-320a mimic/inhibitor transfection. An increased green fluorescence was observed in cells transfected with miR-320a mimic compared to its respective control. Moreover, cells transfected with miR-320a inhibitor exhibited less fluorescence in relation to its control (Fig 3). In addition, *FoxO1* gene expression was evaluated in these cells resulting in an increase in *FoxO1* mRNA levels after miR-320a inhibitor transfection (S6 Fig).

## Discussion

MiR-320a is overexpressed in osteoporotic bone [2], but its role in osteoblastic function was unknown. In the present study, we overexpressed or inhibited miR-320a in hOBs and performed a microarray analysis to reveal the regulated genes and pathways. Additionally, comprehensive functional analyses were carried out in human primary osteoblasts. Microarray results showed a number of key osteoblast genes, together with genes involved in oxidative stress, that were regulated by miR-320a. We also observed an alteration in osteoblast functionality, involving increased proliferation, reduced mineralization, and increased oxidative stress, which might be explained by changes in this gene expression profile.

A total of 38 and 23 genes showed significant expression changes (logFC>1.5) after transfection of miR-320 mimic or inhibitor, respectively, compared to the control transfection samples. No gene was found in both the mimic and inhibitor transfection lists. When logFC was decreased to >1.2, a set of 32 overlapped genes was obtained, although the sign differed between the transfections in 7 of them. As we were dealing with gene expression in primary cells, we observed considerable differences between patients, which precluded obtaining larger logFCs. Another limitation is that microarray assessment of the effects of miRNA-320a cannot discriminate between directly and indirectly regulated genes. Hence, we cannot rule out that many of the altered genes after miR-320a transfection were regulated for indirect pathways. This could explain the differences found between mimic- and inhibitor-regulated genes. Nonetheless, using microarrays allowed us to obtain a general view of the biological pathways affected.

The bioinformatic analysis revealed that pathways involved in the osteoblastic function may be altered after modification of miR-320a cell levels, which had not previously been described. The other enriched pathway (best score) was that of the cellular response to cadmium and zinc ions, mostly by the overexpression of metallothioneins (MTs), which have a role in regulating the zinc pool necessary for bone growth. Zinc has a potent stimulatory effect on osteoblastic bone formation and an inhibitory effect on osteoclastic bone resorption. Therefore, zinc deficiency decreases chondrocyte proliferation and metaphysis heights, along with increased osteoclast density [22]. Apart from the specific function of MTs in homeostasis of essential metals, they also have an antioxidant role against reactive oxygen species (ROS) and protection against DNA damage [23]. These results helped us to focus on further functional studies.

Transfection results of miR-320a in hOB showed that this miRNA led to increase of proliferation but to reduce the ALP activity and mineralization capability, consistent with a loss of osteoblastic differentiation. It is corroborated by the depletion of *HOXA10* and *RUNX2* gene expression after miR-320a overexpression. Moreover, a subtle reduction of *COL1A1* and *BGLAP* (osteocalcin) gene expression was also detected after miR-320a overexpression. These results suggest an explanation for the higher levels of this miRNA detected in fractured osteoporotic bone [2]: overexpression of this miRNA may lead to an impaired mineralization and, consequently, to an osteoporotic phenotype.

It is worth mentioning the different effect between mimic and inhibitor, which could be explained by the microRNA affinity to the gene target and/or the cellular levels of miR-320a. For instance, the action of the miRNA would not be counteracted by its inhibitor if the miRNA effect is small *per se* whereas an overexpression of the miRNA could increase its effect to detectable levels.

Since cellular oxidative stress was the other pathway predicted to be affected by miR-320a, we measured ROS in hOBs. Results consistently showed that all cells transfected with miR-320a mimic had increased ROS levels compared to cells transfected with the control mimic. The opposite effect was observed following miR-320a inhibition, with a reduction of ROS. In primary cells, low ROS levels were observed as a result of normal cell metabolism, and were controlled effectively by the potent cellular antioxidant defense system. The increased ROS levels due to miR-320a transfection may stimulate cell proliferation [24], as well as upregulate MT-1 expression as a response to oxidative stress [25]. Moreover, these increased levels of ROS might contribute to the observed osteoblastic dysfunction [26]. In this context, FoxO1, a transcription factor indispensable for both redox homeostasis and bone metabolism [27], could be an intermediate element in this process since it was significantly increased after miR-320a inhibition. However, the link between increased ROS and osteoblast dysfunction during miR-320a overexpression remains an hypothesis at this stage and needs to be further explored. Nevertheless, it is known that the generation of ROS (including nitric oxide, peroxide ion, etc.) is one of the factors responsible for osteoporosis [28, 29] and this may be one of the mechanisms that can explain the deleterious effect of miR-320a on bone metabolism.

In conclusion, overexpression of miR-320a produces an increase in stress oxidation levels and a reduced mineralization capacity in osteoblastic cells. This disruption of optimal osteoblast function may trigger an osteoporotic phenotype.

## Supporting information

S1 Fig (a) miRIDIAN microRNA Mimic Transfection Control with Dy547 at 100 nM performed in hOBs. Cell nucleus was stained with DAPI. Magnification 20x with the Leica DM IL LED inverted microscope. (b) hOBs were transfected with mimic (100 nM) and inhibitor (400 nM) of miR-320a and the respective miRNA controls. MiRNA levels were measured 48 hours post-transfection by qPCR. Data represent the mean ± SD (n = 2). **p<0.01.
(JPG)

S2 Fig Heat map of differently expressed mRNAs in control mimic vs. mimic samples. Experiments were performed in five hOBs samples. The heat map represents a hierarchical cluster analysis of the differentially expressed mRNAs after a comparison of the control mimic, first five columns, and the miR-320a mimic, five last columns (Control Mimic -Mimic). Each row represents one mRNA and each column, a sample. The mRNA clustering tree is shown at the top of the panel. The color scale illustrates the relative level of the corresponding mRNA expression: red, higher than the reference, and blue, below the reference.
(TIF)

S3 Fig Quantification of gene expression by real time PCR of genes significantly regulated by miR-320a to validate microarray results. (TIF)

S4 Fig Heat map of differentially expressed mRNAs in inhibitor vs. control inhibitor. Experiments were performed in five hOBs samples. The heat map represents a hierarchical cluster analysis of the differentially expressed mRNAs after a comparison between the control inhibitor, first five columns, and the miR-320a inhibitor, five last columns (Inhibitor–Control inhibitor). Each row represents an mRNA and each column, a sample. The mRNA clustering tree is shown at the top of the panel. The color scale illustrates the relative level of the corresponding mRNA expression: red, higher than the reference, and blue, below the reference.
(TIF)

S5 Fig Quantification of gene expression by real time PCR of genes involved in osteoblast differentiation and genes of bone extracellular matrix after miR-320a mimic and inhibitor transfection. (TIF)

S6 Fig Quantification of *FOXO1* gene expression by real time PCR after miR-320a mimic and inhibitor transfection (n = 5). (TIF)
