# Supplementary figures and images for: Pro-osteoporotic miR-320a impairs osteoblast function and induces oxidative stress
Source: PLoS One. 2018 Nov 28;13(11):e0208131. doi: 10.1371/journal.pone.0208131 (PMC6261634; doi:10.1371/journal.pone.0208131)

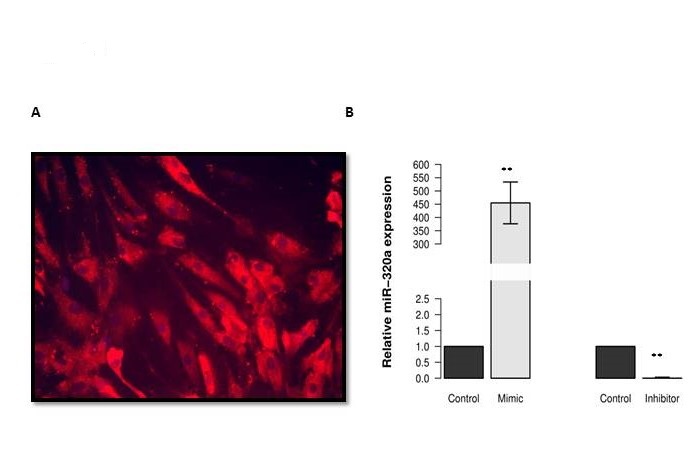

Supplement: S1 Fig — (a) miRIDIAN microRNA Mimic Transfection Control with Dy547 at 100 nM performed in hOBs. Cell nucleus was stained with DAPI. Magnification 20x with the Leica DM IL LED inverted microscope. (b) hOBs were transfected with mimic (100 nM) and inhibitor (400 nM) of miR-320a and the respective miRNA controls. MiRNA levels were measured 48 hours post-transfection by qPCR. Data represent the mean ± SD (n = 2). **p<0.01. (JPG) [file pone.0208131.s001.JPG]

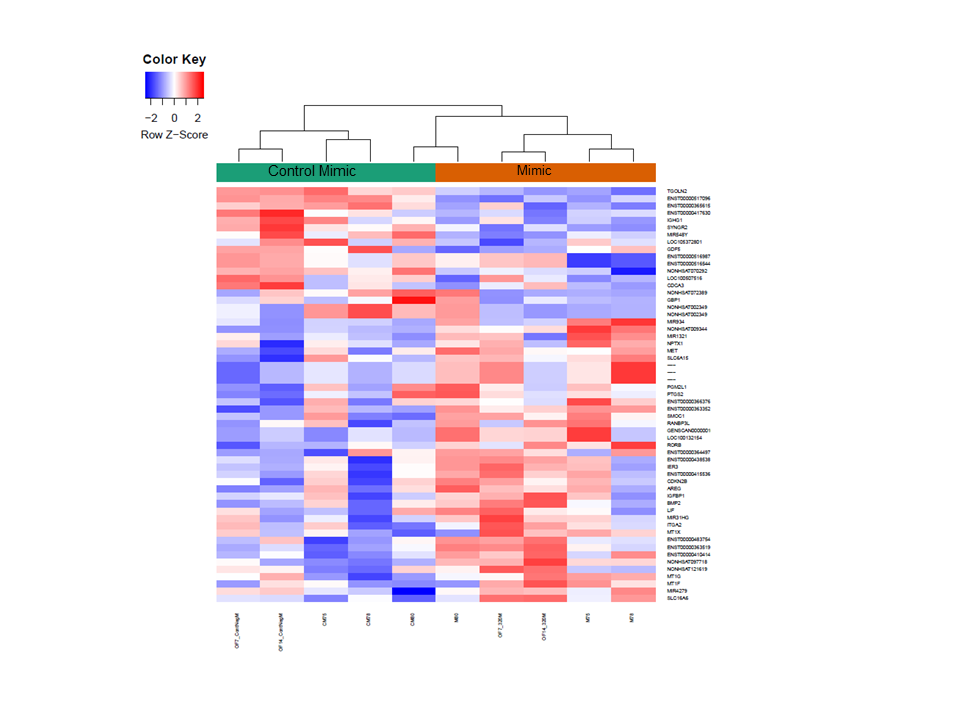

Supplement: S2 Fig — Experiments were performed in five hOBs samples. The heat map represents a hierarchical cluster analysis of the differentially expressed mRNAs after a comparison of the control mimic, first five columns, and the miR-320a mimic, five last columns (Control Mimic -Mimic). Each row represents one mRNA and each column, a sample. The mRNA clustering tree is shown at the top of the panel. The color scale illustrates the relative level of the corresponding mRNA expression: red, higher than the reference, and blue, below the reference. (TIF) [file pone.0208131.s002.tif]

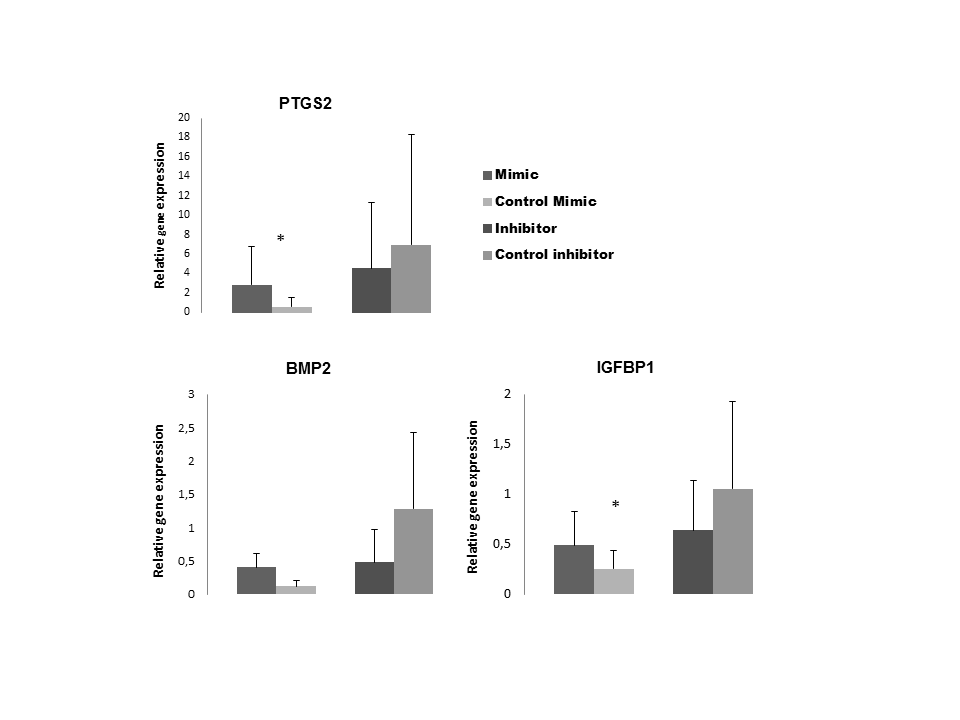

Supplement: S3 Fig — (TIF) [file pone.0208131.s003.tif]

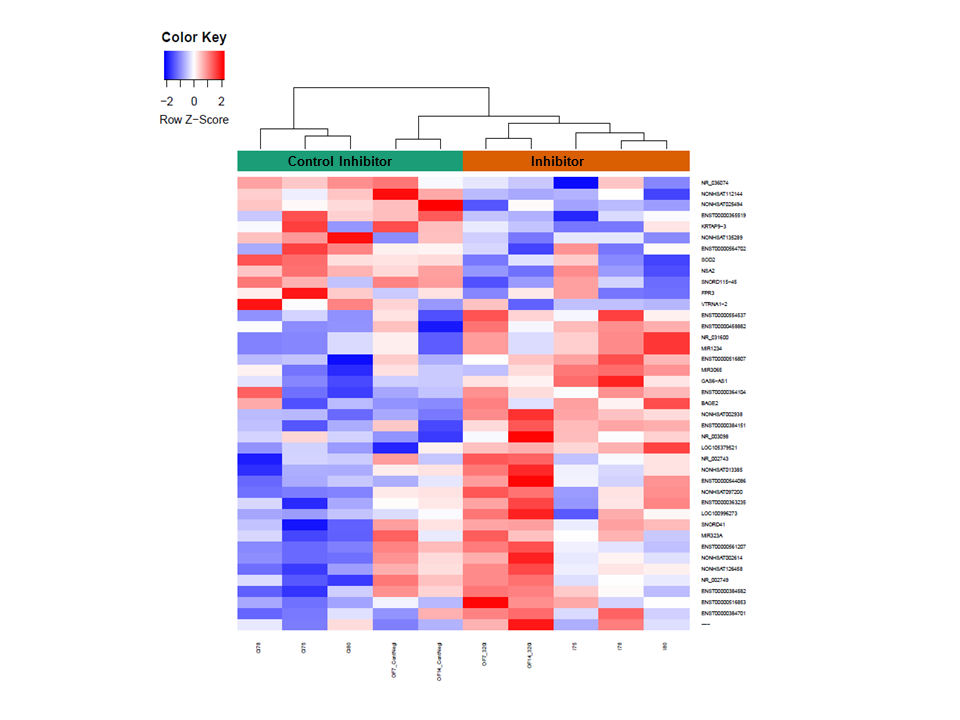

Supplement: S4 Fig — Experiments were performed in five hOBs samples. The heat map represents a hierarchical cluster analysis of the differentially expressed mRNAs after a comparison between the control inhibitor, first five columns, and the miR-320a inhibitor, five last columns (Inhibitor–Control inhibitor). Each row represents an mRNA and each column, a sample. The mRNA clustering tree is shown at the top of the panel. The color scale illustrates the relative level of the corresponding mRNA expression: red, higher than the reference, and blue, below the reference. (TIF) [file pone.0208131.s004.tif]

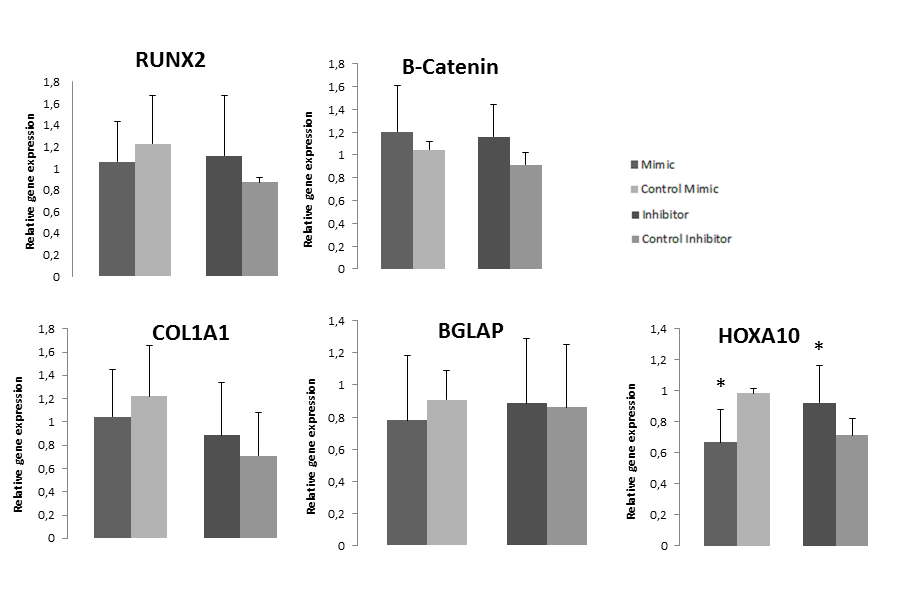

Supplement: S5 Fig — (TIF) [file pone.0208131.s005.tif]

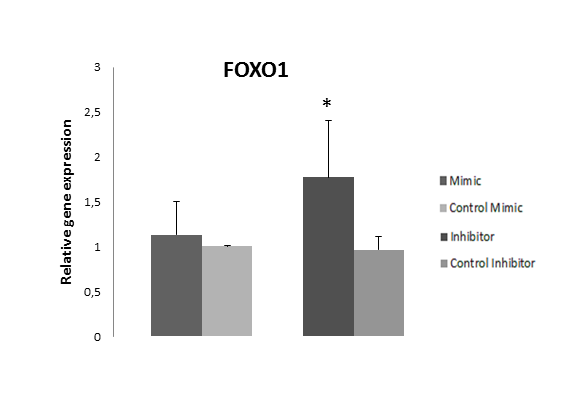

Supplement: S6 Fig — (TIF) [file pone.0208131.s006.tif]
